# Supplementary material for: Tracking SARS-CoV-2 Spike Protein Mutations in the United States (2020/01 – 2021/03) Using a Statistical Learning Strategy
Source: bioRxiv. 2021 Jun 15:2021.06.15.448495. Preprint. [Version 1] doi: 10.1101/2021.06.15.448495 (PMC8219100; doi:10.1101/2021.06.15.448495)
Supplement: 1 [file NIHPP2021.06.15.448495V1-supplement-1.pdf]

bioRxiv preprint doi: https://doi.org/10.1101/2021.06.15.448495; this version posted June 15, 2021. The copyright holder for this preprint (which was not certified by peer review) is the author/funder, who has granted bioRxiv a license to display the preprint in perpetuity. It is made available under aCC-BY 4.0 International license.

Supplementary Material

Materials and Methods

Fig. S1. For all Spike residues with sufficient variation, scatterplots of the maximum proportion (Pmax) of sequences from a given state/territory harboring a mutation at a given amino acid position vs. q-value. Points in red represent residues that meet both criteria for classification as a VRV. Points in black represent residues that do not.

Fig. S2. Temporal patterns of VRVs identified in each state/territory.

Fig. S3. Locally averaged proportions over time for substitutions at 3 AA-subs in a VOI (Y144, F888, V1176) and at 3 AA-subs in a VOC (H69, Y144, K417) that were not detected by the SLS method in states/territories where at least three sequences had a substitution at the designated AA position. AA-sub, amino acid that has been shown to harbor a substitution in a US-circulating VOI or VOC. VOI, variant of interest; VOC, variant of concern.

Fig. S4. Presence of VRVs among all cases in each state/territory. A gray cell means the VRV was not identified in the given case; a black cell means that it was. Both cases and VRVs were clustered by two-way hierarchical cluster analysis.

Table S1. Distribution of the 21,391 VOI/VOC sequences by specific variant and by state/territory.

Table S2. Distribution of the 167,893 SARS-CoV-2 sequences by state/territory and by GISAID submission month, along with state/territory-specific distribution of the 21,391 VOI/VOC sequences that were excluded from the analysis.

Table S3. The 10 identified geo-VRV clusters (TP1 through TP10), based on temporal profiles.

Table S4. Frequencies of the 90 viral residue variants (VRVs) by state/territory, from an unsupervised learning from bi-clustering of all States and VRVs.

bioRxiv preprint doi: https://doi.org/10.1101/2021.06.15.448495; this version posted June 15, 2021. The copyright holder for this preprint (which was not certified by peer review) is the author/funder, who has granted bioRxiv a license to display the preprint in perpetuity. It is made available under aCC-BY 4.0 International license.

Table S5. VRV-haplotypes identified within each state/territory, along with state/territory-specific frequencies. The “positivity” column indicates the proportion of mutations in each haplotype block.

Table S6. Identified haplotypes of pressing VRVs in Washington and New York: frequencies, numbers of VRVs and haplotypic polymorphisms (frequency) in each state.
